# Rabies

**Published:** 1867-08

**Authors:** R. P. Hunt

**Affiliations:** Chicago


### RABIES.

By R. P. Hunt, M.D., Chicago.

Rabies is generally understood as hydrophobia, or the cause which conduces to it. Is there such a disease as hydrophobia? There evidently is. Is not the disease less common than is generally supposed? Is there not a ruthless war unnecessarily waged against the dog, man's fastest, firmest, and most steadfast friend? How many cases of hydrophobia are correctly, authentically reported? Is it not a well-known fact, that the wolf and wild dog generally jump at those portions of the body unprotected by clothing? The domestic dog snaps at the easiest and nearest place, necessarily, through the clothing, and often through the boot, thus wiping off the poisonous saliva, and inserting a clean, unpoisoned fang.

Now, there is a popular theory that dogs suffering from hydrophobia, generally so called, fear water. This may be true, I do not gainsay it; but still, cases are reported, and that on high authority, that dogs pursued and persecuted, under the supposition that they were hydrophobic, have been known to spring into and swim large streams. Watson says, that the fear of water, and the difficulty of imbibing it, is not only a gross error, but one perfectly opposed to fact; not only does thirst exist, according to this author, but it is unquenchable. Mr. Youatt, who, possibly, has more reputation in the veterinary line than any other man, especially in the English language, says, that "all other quadrupeds, with the exception of the horse, drink with increased avidity·" The great John Hunter, who is well known to every tyro in the profession as one of, if not the only, certainly the greatest father of modern surgery, according to Watson (I have no access to his works), is reported as as saying, "that out of twenty-one cases bitten by dogs supposed to be laboring under hydrophobia, only one fell a victim to it." There are many cases of reputed rabies which are certainly not founded on facts. Now, will some of your many readers and correspondents, better informed than myself, advise us whether the great majority of these cases, reported hydrophobic, are not simply cases of hysteria.

We all know that hysteria is not, as its name asserts, specially peculiar to women, and that it is not a mere fancy but a painful disease, and often incurable.

